# Predictor variables of mental health in the Spanish population confined by COVID‐19

**DOI:** 10.1002/brb3.2515

**Published:** 2022-03-11

**Authors:** María Auxiliadora Robles‐Bello, David Sánchez‐Teruel, Nieves Valencia Naranjo, Lorabi Sohaib

**Affiliations:** ^1^ Department of Psychology University of Jaen Jaen Spain; ^2^ Faculty of Psychology University of Granda Granada Spain

**Keywords:** COVID‐19, hope, mental health, pandemic, self‐efficacy

## Abstract

**Background:**

Drastic changes in the lifestyle of individuals have been caused by coronarivus SARS‐CoV‐2 with lethal effects associated with COVID‐19, which acts as a stressor for the population with adverse effects on mental health status. The aim was to identify which sociodemographic variables and psychological factors predict psychological disorders in the general Spanish population.

**Methods:**

The sample consisted of 699 people exposed to a confinement situation, where 402 (57.51%) were women and 297 (42.49%) were men, between 18 and 73 years old (*M* = 27.79; *SD* = 12.68). Different sociodemographic and psychological variables were assessed to see if they predicted levels of anxiety and depression.

**Results:**

The results identify the predictive capacity of some sociodemographic risk variables such as sex (*β* = .144; IC95% = 1.341–3.376) and living with people who are ill with COVID‐19 (*β* = .088; IC95% = 1.157–6.785), as well as protective factors such as self‐efficacy (*β* = −.126; IC95% = −0.282–0.066) and hope (*β* = −.429; IC95% = −0.591–0.408) in mental health. In predicting anxiety levels, self‐efficacy and hope are protective factors, especially when living with people in essential services. In levels of depression, only hope is considered a protective factor in people living with patients infected by COVID‐19.

**Conclusions:**

This study is the first to advance in the understanding of sociodemographic and psychological variables in a Spanish sample subjected to the stressful and traumatic effects of the SARS‐CoV‐2 viral agent.

## Abstract 2

Drastic changes in the lifestyle of individuals have been caused by the coronarivus SARS‐CoV‐2 with lethal effects associated with COVID‐19, which acts as a stressor for the population with adverse effects on mental health status. The aim was to identify which sociodemographic variables and psychological factors predict psychological disorders in the general Spanish population. The results identify the predictive capacity of some socio‐demographic risk variables such as sex and living with people who are ill with COVID‐19, as well as protective factors such as self‐efficacy and hope in mental health. In predicting anxiety levels, self‐efficacy and hope are protective factors, especially when living with people in essential services. In levels of depression, only hope is seen as a protective factor in people living with patients infected by COVID‐19.

## 1 INTRODUCTION

The coronavirus pandemic (COVID‐19) has become a threat to global health (Mahase, 2020; World Health Organization, 2020). Since its identification at the end of 2019, research has been conducted at a rapid pace, focusing on epidemiological aspects (Rothan & Byrareddy, 2020), clinical characteristics (Chen, 2020), and genomic characterization of the COVID‐19 virus (Lu, 2020). There are studies on the mental health of the population exposed to this pandemic (Lenzo et al., 2020; Robles‐Bello et al., 2020; C. Wang et al., 2020), and the results of these studies show that more than half of the exposed population presented moderate‐to‐severe psychological alterations (C. Wang et al., 2020).

According to the World Economic Forum's (2019) Competitiveness Index, Spain is the country with the best health system in the world, along with Singapore, Hong Kong, and Japan. However, the infectious action of COVID‐19 together with these changes (population containment measures) collapsed health system, and a significant number of deaths have represented a stressful and traumatic situation for a high percentage of the Spanish population.

One aspect related to mental health is hope, conceptualized by Snyder (2002) as the perceived ability to plan a course of action towards a goal (pathway), as well as to initiate action and follow the most appropriate paths to success (self‐efficacy). The positive emotions associated with the state of hope are eliminated when the assessment is not only complex but also impossible to deal with developing a state of hopelessness. A second construct that is relevant in this pandemic context is that of general self‐efficacy, conceptualized as the belief in one's ability to adequately manage a wide range of stressors (Konaszewski et al., 2019). Hope and self‐efficacy are related constructs, but present particular characteristics; we can conceive of hope as the perception of the capacity to plan ahead and maintain the motivation to act, if the situation is complex, and almost impossible, while self‐efficacy is the perception of the capacity to be efficient when the situation is complex, but not impossible (Schwarzer & Warner, 2013). Hope and self‐efficacy are directly related to the mental health of individuals (Schotanus‐Dijkstra et al., 2019). More specifically, they have been positively related to psychological well‐being (Pleeging et al., 2019), life satisfaction (Muñoz et al., 2016), and coping with stress (Roesch et al., 2010) and negatively to depressive symptoms (Tehranchi et al., 2018) or anxiety (Siril et al., 2020). When examining other variables that contribute to predicting the state of mental health, attention is paid to various sociodemographic variables identified in previous studies and linked to the COVID‐19 such as age (C. Wang et al., 2020) and sex (C. Wang et al., 2020), type of activity carried out (Liang et al., 2020; C. Wang et al., 2020), care of other people (e.g., minors or elderly), family size or contact with people who are infected or susceptible to greater infection (C. Wang et al., 2020). The hypothesis maintained is the effect on the state of mental health of the participants of both sociodemographic factors linked to the pandemic, as well as the cognitive and emotional capacities of the person. This information can facilitate the planning of possible lines of action aimed at limiting the psychological effects of health crises such as the current one.

To our knowledge, the psychological impact of the COVID‐19 pandemic has not been studied empirically in the Spanish population. Therefore, the aim of this paper would be to identify whether the selected sociodemographic variables and/or psychological strength factors related to motivation for action are involved in predicting levels of anxiety and depression. In the context of this work, it is hypothesized that the general goal of individuals is to cope with the various present and future personal circumstances linked to the COVID‐19 pandemic and that self‐efficacy and hope play a key role in this process.

## 2 METHODS

### 2.1 Participants

A total of 700 people answered the request to participate in the online questionnaire “Emotions in time of crisis” about the psychological state of the general Spanish population in confinement by the COVID‐19. The valid responses were 699, excluding one interviewee due to incoherence in providing his or her sociodemographic data. The criteria for inclusion in the study were: (1) to be 18 years or older and (2) to have completed the questionnaire. The final sample consisted of 699 people, where 402 (57.51%) were women and 297 (42.49%) were men, between 18 and 73 years old (*M* = 27.79; *SD* = 12.68). In 19 cases, a family member presented symptoms of COVID‐19, and in 414 cases, a family member performed some essential services (Table 1).

**TABLE 1:** Description of sociodemographic data of the simple

	*N* (%)	Women, *n* (%)	Men, *n* (%)
Age			
18–28	426 (60.94)	247 (57.98)	179 (42.02)
29–39	132 (18.89)	79 (59.85)	53 (40.15)
40–49	124 (17.74)	66 (53.23)	58 (46.77)
Over 50	17 (2.43)	10 (58.82)	7 (41.18)
Do you work or study?			
Study	255 (36.48)	149 (58.43)	106 (41.57)
Work	359 (51.36)	203 (56.55)	156 (43.45)
Study and work	49 (7.01)	29 (59.18)	20 (40.82)
Retired	14 (2.00)	9 (64.29)	5 (35.71)
Nothing	22 (3.15)	12 (54.55)	10 (45.45)
Number of people confined in the same place			
1	38 (5.44)	21(52.26)	17(47.74)
2	120 (17.17)	75(62.50)	45(37.50)
3	167 (23.89)	102 (61.08)	65(38.92)
4	279 (39.91)	148 (53.05)	131 (46.95)
5	75 (10.73)	45 (60.00)	30 (40.00)
Over 6	20 (2.86)	11 (55.00)	9 (45.00)
Families with children under 14			
Yes	152 (21.75)	89 (58.55)	63 (41.45)
No	547 (78.25)	313 (57.22)	234 (42.78)
Families with people over 60			
Yes	121 (17.31)	78 (64.46)	43 (35.54)
No	578 (82.69)	324 (56.06)	254(43.94)
Presence of COVID‐19 infection			
Yes	19 (2.02)	11 (57.89)	8 (42.11)
No	680 (97.28)	391 (57.50)	289 (42.50)
Relationship with essential service workers			
Yes	414 (59.23)	238 (57.49)	176 (42.51)
No	285 (40.77)	164 (57.54)	121 (42.46)
Total	699 (100)	402 (100)	297 (100)

### 2.2 Instruments

*Sociodemographic data sheet*. Fact sheet designed by the authors for this research with information on gender, age, activity carried out, number of people confined at home, presence of elderly people, presence of under age, living with patients who present symptoms of COVID‐19 infection, and living with essential service workers.

*Hospital Anxiety and Depression (HAD‐14) by Zigmond and Snaith (* *1983* *) in its Spanish version by Herrero et al. (* *2003*). A 14‐item scale was designed for the assessment of anxiety and depression in non‐psychiatric outpatient hospital services. It is a state measure containing two scales, one for anxiety and another for depression. One of its main strengths is the suppression of somatic symptoms, so that it can be assessed independently of the underlying somatic disease. It is a useful instrument validated in our environment and of special interest and relevance in the context of primary care. It presents a subscale of anxiety of seven items and a subscale of depression of seven items in a 4‐point Likert type format, giving the maximum subscale scores of 21 for both depression and anxiety subscales. The questionnaire evaluates the symptoms during the previous week. This scale has a good internal consistency of .90 according to Cronbach's alpha for the full scale; .84 for the depression subscale, and .85 for the anxiety subscale (Herrero et al., 2003). In this study, the alpha on the value of the total inventory was .86, and they were also adequate for the remaining subdimensions (*α_Anxiety_* = .89; *α_Depression_* = .83).

*Hope Herth Index (HHI) of* *Herth (1992)*. The Spanish adaptation by Sánchez‐Teruel et al. (2020) of this scale consists of 12 items with an answer system in a 4‐point Likert scale. Items 3 and 6 are formulated inversely. It is applicable between 16 and 40 years old. It measures hope through three subdimensions: temporality and future; positive disposition and hope; and interconnection. The maximum score is 48 and the minimum is 12. Cronbach's alpha for the Spanish university sample is .89. Total alpha was .69 in this study.

*General Self‐Efficacy Scale‐GSE (* *Baessler & Schwarcer*, *1996*). Adapted to Spanish as Escala de Autoeficacia General [Escala de Autoeficacia General] by Sanjuán‐Suárez et al. (2000). The scale examines general self‐efficacy, i.e., the belief that one's actions are responsible for successful outcomes. The scale includes 10 items with responses on a 4‐point Likert scale. Its score ranges from 10 to 40 points. The internal consistency (Cronbach's alpha) of the Spanish version was .84, and in this study, it was .90.

### 2.3 Procedure

The dissemination of the study and request for participation was carried out between April 15 and 22, 2020. The link https://forms.gle/kAU1sr84uCTHCfMu8 was used for this purpose. The procedure followed was like a “snowball” consisting of contacting the members of the authors' social networks, also requesting that they disseminate it to their own contacts in other groups. The questionnaires were completed through an online survey platform (Google Forms, license from the University of Spain). Previously, the approval of the Ethics Committee of the University of Spain had been requested and obtained (code: ABR.20/4.PRY), which also complies with the principles enshrined in the Declaration of Helsinki. Before completing the survey, the interviewees provided their voluntary and informed consent.

### 2.4 Data analysis

Dependent variables were also self‐reported anxiety and depression symptoms (HADT), anxiety symptoms (HADA), and depression symptoms (HADD). The predictive ability of independent sociodemographic variables (sex, age, activity developed, family members confined together, elderly care, child care, living with ill people with symptoms of COVID‐19 infection and living with essential service workers) as well as those related to psychological abilities (general hope and self‐efficacy) were analyzed by means of multivariate stepwise regression.

To examine the predictive capacity of sociodemographic factors and psychological strengths in the population's mental health status, predictive models were obtained for the global anxiety and depression scale (HADT) as well as for the subcategories anxiety (HADA) and depression (HADD). The sociodemographic variables examined were transformed into “dummy” variables in the case of sex (woman/man); care of the elderly (yes, no) (CuiMay); care of minors (yes, no) (CuiMen); living with persons sickened by COVID‐19 (yes, no) (ConCov), and living with persons in essential services (yes, no) (ConSer). Age was categorized into intervals, while family members (MFam) was considered continuous. The independent variables related to the cognitive variable were general self‐efficacy (GSE) and the emotional variable was hope (HHI). Firstly, sociodemographic variables have been included, and secondly, psychological variables have been included. The appropriateness of the analysis was assessed prior to performing the regression analyses. The results indicated that the assumptions of non‐multicollinearity were met (<5, VIF = 1.00; Kleinbaum et al., 1988), and the tolerance values were between 1 and .62. In addition, there was no autocorrelation in the protective and sociodemographic variables, fulfilling the assumption of independence from error as indicated by the Durwin‐Watson index with a coefficient of nearly two −1.907 in the total scale; 1.851 for the anxiety subscale and 2.012 in the depression subscale, and the results obtained could be generalized to the general population (Chen, 2020).

Goodness‐of‐fit indices were found prior to regression analysis. The statistical analysis of the data was performed with the SPSS statistical package version 24.0.

## 3 RESULTS

In relation to the scale (groups 1, 2, and 3) and gender of the participants, men with anxiety symptoms are 83 (11.87%) of the total (Table 2). Of these, 60.24% are included in Group 1; 27.71% in Group 2, and 12.04% in Group 3. The cases of women with anxiety symptoms are 450 distributed 47.52% in Group 1, 29.11% in Group 2, and 25.55% in Group 3. Men with symptoms of depression are 40: 47.5% with mild symptoms, 42.5% with moderate symptoms, and 10% with severe symptoms. Women who reach the cut‐off point for depression are 140, with 55% of them in Group 1 (mild), 35% in Group 2 (moderate), and 10% in Group 3 (severe).

**TABLE 2:** Frequency and percentages according to symptomatology group and sex

		HADA			HADD		
Group/sex		*F*	%	FG/%	*F*	%	FG/%
G0	M	111	15.88	324/46.41	154	22.03	519/74.3
	W	213	30.47		365	52.21	
G1	M	50	7.15	181/25.89	19	2.72	96/13.75
	W	131	18.74		77	11.02	
G2	M	23	3.29	129/18.45	17	2.43	66/9.45
	W	106	15.16		49	7.01	
G3	M	10	1.43	65/9.30	4	0.57	18/2.57
	W	55	7.87		14	2	

*F*, frequency; FG, frequency per group; Group 0, no alteration; Group 1, mild symptomatology; Group 2, moderate symptomatology; Group 3, severe symptomatology HADA, Hospital Anxiety and Depression Scale (HAD) subscale; HADD, Hospital Anxiety and Depression Scale (HAD) subscale; M, man; W, woman; %, percentage.

The only sociodemographic variables predictive of anxiety and depression (ADH, anxiety, and depression scale) were sex (male/female) and living with COVID‐19 sufferers (ConCov); however, hope and self‐efficacy were protective factors for anxiety and depression in different ways (Table 3). The variance explained by this model 6, referred to depression, was 32.0% (HADD) (*R* ^2 ^= .320; *F*_(3,694)_ = 110.248; *p *< .001), indicating the sex modulation in the prediction of all models (*β_Anxiety_* = .151; IC = .803, 2.064; *p* < .001; *β_Depression_* = .107; IC = .394, 1.470; *p* < .001). Thus, in predicting anxiety (HADA), proximity to people working in essential services (ConSer) (*β_Anxiety_* = .078; IC (95%) = .104, 1.250; *p* < .001) constitutes a risk variable, while in the depression model, the sociodemographic variable of coexistence with people infected by COVID‐19 (ConCov) (*β_Depression_* = .140; IC = 1.884, 4.868; *p* < .001). However, in addition, in predicting anxiety levels, self‐efficacy and hope are protective factors, especially when living with people in essential services. In levels of depression, only hope is considered a protective factor in people living with patients infected by COVID‐19.

**TABLE 3:** Values of the stepwise regression equation for the prediction of measured symptomatology through the total anxiety and depression scale

								IC (95%) *B*	
	*R* ^2^	*F*	*B*		SE	*t*	*β*	L.I.	L.S.
Model 1	.276	266.597**							
HHI			−.613		.038	16.328**	−.526	−.687	−.539
Model 2	.296	147.415**							
Sex			2.375		.522	4.549**	.145	1.350	3.400
HHI			−.602		.037	16.245**	−.517	−.675	−.530
Model 3	.308	103.049**							
Sex			2.267		.520	4.372**	.130	1.246	3.287
HHI			−.510		.047	10.940**	−.438	−.602	−.419
GSE			−.178		.055	3.217**	−.129	−.286	−.069
Model 4 HAD	.312	79.949**							
Sex			2.358		.518	4.550**	.144	1.341	3.376
ConCov			3.971		1.453	2.771*	.088	1.157	6.785
GSE			−.174		.055	3.154*	−.126	−.282	−.066
HHI			−.499		.047	20.707**	−.429	−.591	−.408
Model 5 HADA	.209	46.972							
Sex			1.433		.321	4.465**	.151	.803	2.064
ConSer			.677		.292	2.320*	.078	.104	1.250
HHI			−.209		.029	7.261**	−.311	−.266	−.153
GSE			−.120		.034	3.514*	−.187	−.187	−.053
Model 6 HADD	.320	110.248**							
Sex			.932		.274	3.398**	.107	.394	1.470
ConCov			3.376		.760	4.444**	.140	1.884	4.868
HHI			−.322		.020	16.393**	−.518	−.360	−.283

*B*, non‐standardized coefficient; *β*, result of the regression or beta equation; C.I., confidence intervals; ConCov, living with COVID‐19 sick persons; ConSer, living with essential service persons; *F*, contrast statistics (ANOVA); ns, not significant; g.l., degrees of freedom; GSE, general self‐efficacy; HHI, hope; L.I., lower limit; SE, standard error; S.L. ,upper limit; *R* ^2^, corrected determination coefficient; *t*, predictive variable contrast statistics.

*p < 0.05;**p < 0.01.

## 4 DISCUSSION

The objectives of this work were to identify whether sociodemographic variables and psychosocial factors (self‐efficacy and hope) related to motivation for action, participate in predicting levels of anxiety and depression in the general Spanish population subjected to the COVID‐19 pandemic.

The comparison of the results from this study in relation to the incidence of cases of anxiety (53.64%) indicates that the number of cases reaching the cut‐off point is higher than that found by C. Wang et al. (2020) and Y. Wang et al. (2020). The percentages are also generally higher in the groups with mild (25.89%) or moderate (18.45%) symptoms. In addition, C. Wang et al. (2020) did not identify cases in the severe anxiety group (Group 3), although they have been identified in this study (9.30%). The categorization of depression offers more comparable results between studies, although the frequency of cases (25.78%) in the moderate and severe groups is higher than reported by C. Wang et al. (2020) and lower when compared at these levels with the data from Y. Wang et al. (2020). The data were obtained between April 15 and 22, approximately 4 weeks after the declaration of the State of Alarm in Spain, while in the investigations in China, it was in the following 2 weeks. These different periods may be a factor contributing to the difference in anxiety levels.

Another relevant question regarding the scoring system in the sample of participants is to examine the proportion of men and women in each of the anxiety or depression groups. The data suggest that in men mild symptoms of anxiety (60.24%) but more severe symptoms of depression (moderate and severe) predominate (52.5%). If we look at the group of women, the results indicate that in them more severe symptoms of anxiety (moderate and severe) predominate (52.66%) and mild symptoms of depression (47.53%). This set of results partially coincides with those obtained by Liang et al. (2020) for men, but only when the symptoms are severe.

Regarding the factors participating in the prediction of mental health status examined through anxiety and depression scale, sociodemographic variables have been proposed as factors linked to the effects of the COVID‐19 pandemic (Liang et al., 2020; C. Wang et al., 2020). The psychological variables were hope and general self‐efficacy. The score on the global anxiety and depression scale was predicted by the sex of the participants and by direct contact with people suffering from COVID‐19. Attention to the level of hope and self‐efficacy increases the explained variance (*R* ^2 ^= 31.2%). The analysis of the weights assigned to each of the factors ratifies the contribution (greater weight) of hope as a protective factor in relation to the sociodemographic factors involved. The weight assigned to self‐efficacy is lower in depression and higher in anxiety, but of similar magnitude to the influence of sex and higher than living with patients with COVID‐19. Similar conclusions are reached in the prediction of scores in anxiety and depression.

The relationship between anxiety, depression, and sex is complex and further mediated by age, with usually more anxiety symptoms in women in the early stages of life until the incidence of sex progressively balances out at later ages (Curran et al., 2020). The inclusion of sex as a predictor of anxiety was also obtained by C. Wang et al. (2020) although not in the prediction of depression scores. Another sociodemographic element in the prediction models is the contact maintained with other people. In this paper, a distinction is made between contact with people who are ill with COVID‐19 and people who are at greater risk of contracting the illness (essential services personnel). The first of these factors is involved in predicting depression and the second in predicting anxiety. In contact with a sick person, the situation is threatening and uncontrollable, while contact with essential personnel is also assessed as threatening but more controllable. The perception of uncontrollability has traditionally been linked to depressive states. Living with potential patients also plays a role in predicting distress (total scale of anxiety and depression). In this prediction, the assessment of the uncontrollability of the situation is likely to extend to concerns about contagion among family members (C. Wang et al., 2020).

Moreover, noteworthy in this study is the exclusion of other sociodemographic factors, such as the occupation of the participants or factors considered linked to that state of mental health, such as care of the elderly, care of minors, or the number of family members cared for during confinement. Its absence in the regression models is linked to the contribution that the psychological variables of the individuals have in these models, supporting the idea that a good level of motivation for acting in difficult situations can counteract the influence of these sociodemographic factors.

Hope refers to the individual's expectation that he or she can cope with difficult situations, identifying ways of acting, and feeling motivated to do so, while self‐efficacy relates to the expectation of being effective in implementing action strategies. These two factors contribute significantly to the prediction of the level of general distress as well as anxiety in a negative relationship. The relationship between hope and anxiety has been well established in the previous literature (Gallagher et al., 2020) as well as between anxiety and self‐efficacy (Ng & Lovibond, 2019). A low level of hope contributed to the prediction of the level of depression (Robles‐Bello et al., 2020).

The important contribution of these psychological variables of the individual in the prediction of the level of distress, as well as of anxiety and depression, is considered a relevant data because it opens the perspective for improvement of the state of mental health through intervention programs focused on the psychological strengthening of the population. The suggestions for short and/or low cost temporal and economic interventions (Varker et al., 2019) are interesting. Their results indicate the effectiveness of interventions, especially when they involve more direct contact with the therapist. Another possible line of work, more directed towards less serious cases, is that followed by Schotanus‐Dijkstra et al. (2019), with an intervention supported and guided by a therapist but with strong support of self‐help materials.

This study has some limitations. On the one hand, we used a cross‐sectional design, which cannot provide strong evidence of causality. Secondly, this study used self‐reported questionnaires, which have problems of subjectivity. Furthermore, due to snowball sampling, these findings may not represent the entire Spanish population, although the number of participants can be considered significant (Centre for Sociological Studies, 2020). In addition, more women took part than men. Female participation over male participation is common in psychological research, which may be explained by the greater frequency with which women actively face problems and make more requests for help than men (Liddon et al., 2017). This would be an area for improvement in future research.

## 5 CONCLUSIONS

Coping with a traumatic/stressful experience, such as that associated with a pandemic by a highly infectious agent such as COVID‐19, is complex for individuals. Its effects depend on the circumstances in which we have to deal with it, and the personal resources available to us are involved in this process in a relevant way. The contribution of hope and self‐efficacy to the maintenance and/or improvement of the state of mental health highlights avenues of action with a twofold objective. We consider that the arrival of new pandemics associated with the high globalization experienced in the planet, which promotes the spread of infectious agents in different countries, is foreseeable. This forecast suggests the need for countries to be prepared for the arrival of new pandemics. One form of preparation, we believe, should include the psychological strengthening of their population.

## AUTHOR CONTRIBUTIONS

All authors have contributed equally. The authors approved the final version of the manuscript.

## CONFLICT OF INTEREST

The authors declare that they have no competing interests.

## Data Availability

The dataset generated and analyzed for this study is not publicly available due to the restrictions claimed in the document of the research permission and ethical approval.
